# Direction of Arrival Estimation of GPS Narrowband Jammers Using High-Resolution Techniques

**DOI:** 10.3390/s19245532

**Published:** 2019-12-14

**Authors:** Mohamed Moussa, Abdalla Osman, Mohamed Tamazin, Michael J. Korenberg, Aboelmagd Noureldin

**Affiliations:** 1 Electrical and Computer Engineering Department, Queen’s University, Kingston, ON K7L 3N6, Canada; mohamed.moussa@queensu.ca (M.M.); korenber@queensu.ca (M.J.K.); 2 Electronics and Communications Engineering Department, Arab Academy for Science, Technology and Maritime Transport (AASTMT), Alexandria, Egypt; 3 Electrical and Computer Engineering, Royal Military College of Canada, Kingston, ON K7K 7B4, Canada; osman.a.m7@gmail.com (A.O.); Aboelmagd.Noureldin@rmc.ca (A.N.)

**Keywords:** GPS, jamming detection, array processing, fast orthogonal search

## Abstract

GPS jamming is a considerable threat to applications that rely on GPS position, velocity, and time. Jamming detection is the first step in the mitigation process. The direction of arrival (DOA) estimation of jamming signals is affected by resolution. In the presence of multiple jamming sources whose spatial separation is very narrow, an incorrect number of jammers can be detected. Consequently, mitigation will be affected. The ultimate objective of this research is to enhance GPS receivers’ anti-jamming abilities. This research proposes an enhancement to the anti-jamming detection ability of GPS receivers that are equipped with a uniform linear array (ULA) and uniform circular array (UCA). The proposed array processing method utilizes fast orthogonal search (FOS) to target the accurate detection of the DOA of both single and multiple in-band CW jammers. Its performance is compared to the classical method and MUSIC. GPS signals obtained from a Spirent GSS6700 simulator and CW jamming signals were used. The proposed method produces a threefold advantage, higher accuracy DOA estimates, amplitudes, and a correct number of jammers. Therefore, the anti-jamming process can be significantly improved by limiting the erroneous spatial attenuation of GPS signals arriving from an angle close to the jammer.

## 1. Introduction

A GPS signal is at a disadvantage because of its low signal power, which is typically between –125 dBm and –130 dBm at the earth’s surface and is therefore highly susceptible to intentional and non-intentional interferences. GPS is relied upon in many critical infrastructure sectors [1]. Such an example is the civil aviation sector, which is characterized by a safety-critical nature that imposes stringent requirements on the availability of the GPS solution. In this case, GPS is used to help land aircraft using ground-based augmentation systems (GBAS). They are steadily deployed, but their operation can be disrupted when interference and jamming signals are in an airport’s vicinity. A clear example of the threat is Newark Liberty International Airport, where the GBAS system’s operation was affected due to GPS jamming using an illegal personal privacy device (PPD) [2]. When interference is present, detecting it is a crucial first step toward its mitigation.

In order to detect and mitigate interference, many authors have proposed solutions that exploit the time domain, frequency domain, space domains, or combinations of the fields. In the time domain, pulse blanking is commonly used, whereas, in the frequency domain, adaptive filtering is used [3,4,5]. Time-domain pulse blanking and frequency excision introduce a set of drawbacks such as distorted correlation peaks, acquisition problems, and tracking performance degradation. When spatial mitigation is used, the drawbacks mentioned above will no longer be evident.

In the space domain, adaptive antenna arrays (smart antennas) that employ spatial processing are used. Accordingly, several advantageous and unique capabilities are introduced, for example, the direction of arrival (DOA) estimation, beam-steering, and null-steering. Once the DOA estimates are computed, interference mitigation can be achieved using null steering by placing nulls in the detected direction of interference [4,6,7,8]. On the other hand, beam-steering directs the main lobe toward the direction of arrival of the GPS signal [9].

Therefore, smart antennas continue to attract interest in the field of GPS interference detection and mitigation due to their superiority compared to single-antenna receivers, when dealing with intentional and non-intentional interferences. The interference mitigation capability of smart antennas is mainly based on the accuracy of the DOA estimation of interference sources [4,10,11]. For reliable operation, GPS interference mitigation using smart antennas should be capable of detecting multiple interference sources arriving at different DOAs. Conventional beam-forming methods provide DOA estimation for interference sources with limited resolution. This degrades the spatial filtering capability of smart antennas when the jamming signal is closely spaced to the DOA of the GPS signal. Accordingly, high-resolution DOA estimation methods are more suitable for interference mitigation using smart antennas. Various high-resolution DOA estimation methods have been developed, including MUltiple SIgnal Classification (MUSIC) [12,13] and estimation of signal parameters via rotational invariance techniques (ESPRIT) [14]. However, the performance of these methods degrades severely as the coherence between jamming signals increases [15].

This paper aims to propose a new high-resolution DOA estimation method of jammers in order to provide smart antennas with the most accurate estimate of the jammers’ DoAs such that beam-steering and null-steering processors nullify the jammer signals without erroneously nullifying parts of the GPS signal, thereby decreasing its effectiveness. The proposed method was designed to operate in the precorrelation section of GPS receivers, where mitigation is more effective. It is based on the orthogonal search (OS) technique [16] and creates bases for the steering function. The coefficients of the steering function are determined via a unique orthogonal search method that provides high-accuracy DOA estimation. The proposed method can enhance anti-jamming techniques that utilize antenna arrays, specifically in situations where the DOA of jamming signals is very close to the DOA of the GPS signal. Hence, it can enhance the null-steering process and avoid a reduction in GPS signal levels while rejecting jamming signals. Furthermore, the high-resolution capabilities of the proposed method can resolve jamming signals arriving at very close DOAs and hence improves the overall performance of the anti-jamming method. 

The proposed method is compared to classical DOA estimation and MUSIC using GPS L1 signals obtained from a Spirent 6700 simulator; the arrays utilized are uniform linear array (ULA) and uniform circular array (UCA) arrays. The jamming signals were simulated as CW sources originating from different directions. The performance of the investigated methods is evaluated in the case of single and multiple interference signals. Our results showed that the proposed method introduces a significant enhancement to GPS jamming detection and hence can improve overall system performance.

## 2. DOA Estimation Methods

Spatial domain detection and mitigation is a mature field where many DOA estimation methods have been proposed. Classical beam-forming, also known as conventional beam-forming, [17] represents an earlier approach to DOA estimation; the spatial spectrum is scanned using predetermined angular steps in search of the direction angle that corresponds to the highest spectral power. The drawback of this method is its inability to form sharp peaks unless a large number of array elements are used. Consequently, the method suffers from a limited ability to resolve closely spaced sources [18].

Capon’s beamformer, also known as the minimum variance distortionless response (MVDR), attempts to improve the drawbacks associated with the classical method and yield a significant improvement [19]. The output power is minimized while constraining the gain in the necessary direction to unity. The MVDR method requires an additional matrix inversion compared to the classical method. Fortunately, it outperforms the conventional method in most cases. The disadvantages of the MVDR method are the necessary other computations and the failure to estimate the DOAs of highly correlated signals [20].

Advances in the field of direction-finding lead to the development of high-resolution DOA estimation methods that utilize the signal subspace. By performing Eigenanalysis on the spatial covariance matrix, the signal and noise subspaces are generated. MUSIC is among these subspace techniques [21]. MUSIC showed that the steering vectors associated with the received signals are found in the signal subspace. A search throughout the possible steering vectors is conducted; the ones that are orthogonal to the noise subspace are designated as desired signals. However, errors arise in real scenarios, since full orthogonality is challenging to achieve due to errors in the estimation of the noise subspace. The MUSIC spectrum generates a tremendous value when a match between the generated steering vector and the actual DOA occurs. The disadvantage of MUSIC is that it is not able to identify DOAs of correlated signals on its own; the received signal must undergo a preprocessing technique called spatial smoothing [22]. In addition, it is computationally expensive, sensitive to noise, and must have prior knowledge of the number of signals it is looking for. Several variants of the method have been proposed to improve its performance [23].

The estimation of signal parameters via rotational invariance technique (ESPRIT) algorithm was proposed by [24]. It is computationally efficient and more robust if compared to MUSIC as it does not search the entire spectrum, but a significant drawback is an incompatibility with all array geometries, as it was designed for uniform linear arrays (ULA). The method has been extended to include multidimensional arrays, and many versions have been produced [24,25].

Generally, spatial signal processing using antenna arrays is considered one of the most effective techniques for narrowband interference detection and primarily suppression [7,8]. Antenna arrays enable interference mitigation using null steering, beam steering, or a combination of both. This is achieved using adaptive beam-forming and high-resolution DOA estimation methods [26]. The obtained DOA estimates are utilized to produce nulls in the direction of interfering signals, and if beam steering is available, steer the main beam toward the desired GPS signal.

Various DOA estimation algorithms have been developed for array signal processing applications. The selection of the DOA estimation algorithm is a crucial element of adaptive antenna array design. It directly affects null steering and beam-forming processes.

The DOA estimation techniques that are predominantly used are those that are based on classical DOA, Capon, and MUSIC algorithms [26]. It has been reported that Eigen-decomposition based methods such as MUSIC have high-resolution DOA estimation performance [26]. These methods are based on exploiting the Eigenstructure of the input covariance matrix. Applying MUSIC to GPS anti-jamming has shown to provide a significant enhancement to the overall system performance [27]. However, the performance of MUSIC is limited by the coherence of the jamming sources [28].

## 3. DOA Estimation Using Fast Orthogonal Search (FOS)

This research studies the application of FOS in the DOA estimation of jamming signals [5]. FOS is a highly efficient general-purpose modeling method that has been used in several applications [29]. This method is a modification [15] of an original OS algorithm, which constructs a functional expansion of an input data using an arbitrary set of non-orthogonal candidate functions. The functional expansion of the input signal in terms of the arbitrary candidate functions is given by:

(1) $$y\left(n\right)\;=\;\overset{M}{\underset{m=0}{\sum }}a_{m}{\;p}_{m}\left(n\right)\;+\;e\left(n\right)$$

where n is the sample index, ${\left\{a_{m}\right\}}_{m\;=\;0}^{M}$ are the weights of the functional expansion, and e(n) is the modeling error. The signal arriving at the antenna array is generally composed of complex sinusoidal signals and noise. The signal received at the *n^th^* sensor at time $t_{i}$ from S sources is defined by [30]:

(2) $$y_{m}\left(t_{i}\right)\;=\overset{\;S\;-\;1}{\underset{k\;=\;0}{\sum }}P_{k}\left(t_{i}\right)\exp\left(j2{\pi f}_{k}\left(t_{i}\;-{\;\tau }_{m}\left(\theta_{k}\right)\right)\right)\;+\;e\left(t_{i}\right)$$

where i = 0, …,T − 1 is the signal time index, m = 0,…, M − 1 is the sensor index number, k = 0,⋯, S − 1 is the signal index number, $\theta_{k}$ is the DOA of the signal from k^th^ source, $P_{k}$ is the complex amplitude of the $k^{\mathrm{th}}$ source $P_{k}\left(t_{i}\right)\;=\;\left|P_{k}\left(t_{i}\right)\right|\exp\left(j\varphi \left(t_{i}\right)\right)$, $\varphi \left(t_{i}\right)$ is the phase of the $k^{\mathrm{th}}$ signal at its source, and e $\left(t_{i}\right)$ is the modeling error. For L real signals, S = 2 L and the complex amplitudes are conjugate pairs (i.e., ${\;P}_{k\;+\;1}\;={\;P}_{k}^{*}$ and ${\;f}_{k\;+\;1}\;=\;-f_{k}$). The reference is taken at the first element; thus, $\tau_{0}\left(\theta_{k}\right)\;=\;0$.

The following model can represent the received signal at the antenna array:

(3) $$y\left(n\right)=\overset{2L-1}{\underset{k=0}{\sum }}a_{k}\left(\theta_{k}\right)s_{k}\left(n\right)+e\left(n\right)$$

where $a_{k}\left(\theta_{k}\right)$ is the M× 1 antenna array steering vector for the k^th^ source and $s_{k}$ is the k^th^ source signal received by the antenna array. For real signals, each candidate is represented by its amplitude, sinusoidal function (sin or cos), and phase. In order to estimate the amplitude and phase of each candidate, the fitting process is carried by selecting pairs of sin and cos functions for DOAs. Hence, for L DOAs, we have 2L-1 candidate functions.

OS creates an orthogonal basis set [31] for the steering vectors using the Gram–Schmidt procedure. This set forms a model equivalent to Equation (3), which is defined by:

(4) $$y\left(n\right)=\overset{2L-1}{\underset{k=0}{\sum }}w_{k}\left(\theta_{0},\theta_{1},\cdots ,\theta_{r}\right)g_{k}\left(n\right)+e\left(n\right)$$

where r=k/2 for k even values, r=(k−1)/2 for k odd values, $w_{i}^{*}w_{j}=0$ for i≠j (* denotes complex-conjugate transpose), and $\left\{g_{k}\left(n\right)\right\}$ are the coefficients of the orthogonal basis vectors.

The set of orthogonal basis vectors $\left\{w_{k}\right\}$ is obtained from the candidate functions $\left\{a_{k}\left(\theta_{k}\right)\right\}$ according to the following formula:

(5) $$w_{k}=a_{0}-\overset{k-1}{\underset{i=0}{\sum }}\alpha_{\mathrm{ki}}w_{i}$$

where $\alpha_{\mathrm{ki}}=\frac{{w_{i}}^{*}a_{k}}{{w_{i}}^{*}w_{i}}$ and $w_{0}=a_{0}$.

The coefficients $g_{k}\left(n\right)$ are estimated using the following formula:

(6) $$g_{k}\left(n\right)=\frac{{w_{k}}^{*}\;y\left(n\right)}{{w_{k}}^{*}w_{k}}.$$

This formula was derived to minimize the mean square error (MSE) between the observations vector y (n) and the orthogonal model. The total MSE ($\bar{\varepsilon^{2}})$ is defined as:

(7) $$\bar{\varepsilon^{2}}=\frac{1}{T}\overset{T-1}{\underset{n=0}{\sum }}\frac{1}{M}∥y\left(n\right)-\overset{S-1}{\underset{k=0}{\sum }}g_{k}\left(n\right)w_{k}{∥}^{2}$$

$$\;=\frac{1}{\mathrm{MT}}\overset{T-1}{\underset{n=0}{\sum }}y^{*}\left(n\right)y\left(n\right)-\overset{S-1}{\underset{k=0}{\sum }}Q_{k}\;$$

where $Q_{k}$ represents the reduction introduced to the MSE by adding the candidate basis vector $\hat{w_{k}}$ to the model and defined by the following equation:

(8) $$Q_{k}=\frac{1}{\mathrm{MT}}\sum_{n=0}^{T-1}\frac{{\left[{{\;w}_{k}}^{*}y\left(n\right)\right]}^{2}}{{w_{k}}^{*}w_{k}}=\frac{{w_{k}}^{H}w_{k}}{\mathrm{MT}}\sum_{n=0}^{T-1}g_{k}\left(n\right).$$

Equations (5) and (8) indicate that the calculation of $Q_{k}$ involves the correlations of $w_{k}$ with themselves, the steering vectors $a_{k}$, and the data  y(n). The calculation of $Q_{k}$ involves the correlations of $w_{k}$ with themselves; the steering vectors $a_{k}$ and y(n) are the dates. Therefore, the orthogonal search can discard the need to create the orthogonal basis vectors ${\;w}_{k}$ explicitly.

This observation was used in [29,32,33] to accelerate the OS algorithm by discarding the explicit creation of the orthogonal basis vectors $w_{k}$. Accordingly, this approach was named FOS, which stands for fast orthogonal search [29,32,33]. FOS calculates the correlations mentioned above using Cholesky factorization. It starts by creating a variable $D_{\mathrm{km}}$, which represents the correlation between the candidate and the steering vector. Thus, $D_{\mathrm{km}}$ is defined by:

(9) $$D_{\mathrm{km}}=w_{m}a_{k}=a_{m}^{*}a_{k}\;-\overset{m-1}{\underset{j=0}{\sum }}{{\;\alpha }_{\mathrm{mj}}}^{*}{\;D}_{\mathrm{kj}}$$

where m=1…k and ${\;\alpha }_{\mathrm{kj}}=\frac{D_{\mathrm{kj}}\;}{D_{\mathrm{jj}}\;}\;$.

The correlation between the candidate basis vector ${\;w}_{k}$ and the observations vector y (n) is denoted by $C_{\mathrm{nk}}$ and defined by:

(10) $$C_{\mathrm{nk}}={{\;w}_{k}}^{*}y\left(n\right)={{\;a}_{k}}^{*}y\left(n\right)-\sum_{j=0}^{k-1}{{\;\alpha }_{\mathrm{kj}}}^{*}{\;C}_{\mathrm{nj}}.$$

Consequently, the coefficients of orthogonal basis vectors $g_{k}\;\left(n\right)$ and the amount of reduction in MSE $Q_{k}$ are expressed by:

(11) $$g_{k}\;\left(n\right)=\frac{C_{\mathrm{nk}}}{D_{\mathrm{kk}}}$$

(12) $$Q_{k}=\sum_{n=0}^{T-1}\frac{{C_{\mathrm{nk}}}^{2}}{{\mathrm{MTD}}_{\mathrm{kk}}}\;.$$

The derivation of Equations (11) and (12) are based on the properties of orthogonal basis vectors $w_{k},$ which imply that ${\;w}_{k}^{*}w_{k}=w_{k}^{*}a_{k}$. Additionally, given that $w_{0}=a_{0}$, the initial values for $D_{\mathrm{km}}$ and $C_{\mathrm{nk}}$ are defined by the following:

(13) $$D_{m0}=a_{0}^{*}a_{m}\;\mathrm{and}\;C_{n0}=a_{0}^{*}y\left(n\right).$$

FOS constructs the model defined by Equation (4) by adding the candidate functions corresponding to DOA one at a time. The selection of candidate functions corresponding to an estimate ${\hat{\theta }}_{k}$ is based on the assessment of a set of parameters to provide the value that yields maximum MSE reduction $Q_{k}$. Since a real signal is formed from accumulated sine and cosine components, the MSE reduction contributed by a single candidate parameter can be represented by the sum of the MSE reductions induced by the two corresponding steering vectors of that candidate (i.e., $Q_{k}$ and $Q_{k+1}$). Thus, the selected estimate ${\hat{\theta }}_{k}$ is defined by:

(14) $${\hat{\theta }}_{k}=\arg\left[\max\left(Q_{k}+Q_{k+1}\right)\right].$$

Once $\theta_{k}$ is selected, the two terms $g_{k}\left(n\right)w_{k}$ and $\;g_{k+1}\;\left(n\right)w_{k+1}$ are added to the constructed model. This process is repeated until L parameter estimates are chosen. The values of the signal coefficients $s_{k}\left(n\right)$ are obtained recursively using the following formula:

(15) $${\hat{s}}_{k}\left(n\right)=\overset{S-1}{\underset{m=k}{\sum }}g_{m}\left(n\right)v_{m}$$

where $v_{k}=1$ and ${\;v}_{m}=-\overset{m-1}{\underset{j=k}{\sum }}{\;\alpha }_{\mathrm{mj}}v_{j},\;m=k+1,\;k+2,\ldots ,S-1$.

### 3.1. Stopping Conditions for the FOS Algorithm

The FOS algorithm can be stopped using one of the following criteria:

- Reaching a predefined maximum number of terms to be fitted. This requires the knowledge of the number of narrowband interference signals impinging upon the antenna array. However, the orthogonal search approaches may be combined with any of several statistical criteria to determine when to stop adding terms to the model, thus providing an estimate of the number of signals [30].
- Reaching a predefined threshold for MSE reduction. This criterion is achieved when the ratio of MSE to the mean squared value of the input signal is below a predefined threshold. Accordingly, the limitation of knowing the number of expected interference signals is waived. However, it may lead to an increase in processing time. Thus, this ratio should be carefully selected to avoid excessive processing time.
- When adding another term to the model, the MSE reduction is less than the MSE reduction that would be gained if white Gaussian noise was added.

### 3.2. FOS Complexity

FOS necessitates floating-point operations of the order of $\mathrm{CMT}\;+{\;\mathrm{CL}}^{2},$ where C is the number of candidate steering vectors searched [33]. Moreover, if the elements of the array (and therefore, the data samples) are not equally spaced, FOS will require a higher order, which becomes $\mathrm{CMT}\;+{\;\mathrm{CL}}^{2}$ + CML [30].

#### Candidate Function Selection for the FOS Algorithm

The process of candidate function selection plays a crucial role in the FOS algorithm. In this research, the selected candidate functions are pairs of sine and cosine functions corresponding to the DOAs of the search domain. Accordingly, the chosen candidate functions for a ULA and UCA using the model shown in Equation (3) are given by [33]:

(16) $$a_{k}\left(\theta_{k}\right)=\left\{a_{k}\left(m,\theta_{k}\right)\right\}$$

where $\theta_{k}$ is the DOA of the $k^{\mathrm{th}}$ source signal.

When $\theta_{k}$ is measured from the line of the antenna array, the following candidate functions are used

(17) $$a_{k}\left(m,\theta_{k}\right)=\cos\left(2m\pi \left(\frac{d}{\lambda }\right){\cos\theta }_{k}\right)\;a_{k+1}\left(m,\theta_{k}\right)=\sin\left(2m\pi \left(\frac{d}{\lambda }\right){\cos\theta }_{k}\right)\;.$$

On the other hand, when $\theta_{k}$ is measured from the line perpendicular to the antenna array, Equation (18), which is shown below, is utilized:

(18) $$a_{k}\left(m,\theta_{k}\right)=\cos\left(2m\pi \left(\frac{d}{\lambda }\right)\sin(\theta_{k}\right))\;a_{k+1}\left(m,\theta_{k}\right)=\sin\left(2m\pi \left(\frac{d}{\lambda }\right)\sin\left(\theta_{k}\right)\right)\;$$

where m=0, 1…, M−1, i=0,1…, T−1d is the spacing between elements, and λ is the wavelength of the received signal.

The coefficients $s_{k}\left(n\right)$ are given by [33]:

(19) $$s_{k}=\sqrt{2P_{r}}\;\cos\left({\pi t}_{i}+\varphi_{r}\left(t_{i}\right)\right)\;s_{k+1}=\sqrt{2P_{r}}\;\sin\left({\pi t}_{i}+\varphi_{r}\left(t_{i}\right)\right)$$

where $P_{r}$ and $\varphi_{r}\left(t_{i}\right)$ are the power and phase of the $r^{\mathrm{th}}$ source, respectively.

## 4. Antenna Array Design

In the following sections, the array design for the ULA and UCA arrays will be studied from a perspective that will ultimately serve the goal of DOA estimation. Therefore, in order to adhere to the common literature notations, the total number of elements in an array will be referred to as N elements, while n will represent the index of an element in the array and n=0, 1, …, N−1.

### 4.1. Uniform Linear Array

**Figure 1:**
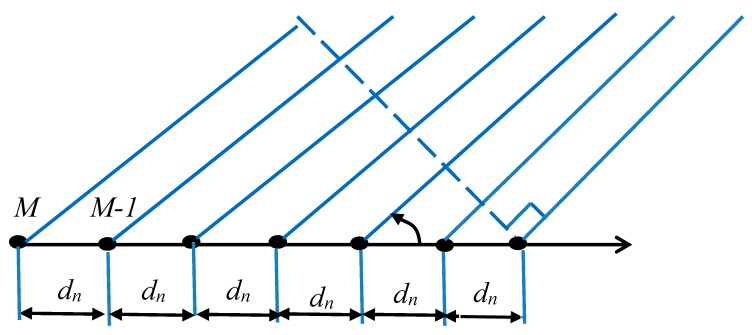
Uniform linear array.

The ULA is capable of filtering the electromagnetic environment in which it operates, based on the location of the signals in its vicinity. Array geometry has a significant effect on the DOA estimation abilities of antenna arrays. In this study [5], the ULA shown in Figure 1 was considered.

For the general ULA showed in Figure 1 with inter-element spacing and dn and M identical elements, the signal received by the antenna array is given by:

(20) $$y\left(t\right)=s\left(t\right)\left(\overset{M-1}{\underset{n=0}{\sum }}e^{-\mathrm{jk}.d_{n}}\right)$$

where s(t) is the transmitted signal.

The quantity in parenthesis is known as the array factor (AF). This factoring is regularly termed pattern multiplication. It can be utilized when the identical array elements are oriented in the same direction. Moreover, the radiation pattern of the array is the result of the multiplication of the element radiation pattern and array factor.

When the elements of the ULA are all located on the z-axis, $\mathrm{dn}=\left(0,0,z_{n}\right).$ Then, the AF becomes:

(21) $$\mathrm{AF}=\left(\overset{M-1}{\underset{n=0}{\sum }}e^{-j\frac{2\pi }{\lambda }z_{n}\cos\theta }\right)$$

where n=0,…, M−1, and θ is the direction of arrival of the received signal.

For an M-element, ULA let $\mathrm{dn}=\left(0,0,\frac{n\lambda }{2}\right)$. Hence, the AF becomes:

(22) $$\mathrm{AF}=\sum_{n=0}^{M-1}e^{-\mathrm{jn}\pi \cos\theta }.$$

The array factor formula can be simplified using the following identity [34]

(23) $$\sum_{n=0}^{M-1}c^{n}=\frac{1-c^{M}}{1-c}.$$

Therefore, the array factor for ULA is given by:

(24) $$\mathrm{AF}=\frac{1-e^{-\mathrm{jM}\pi \cos\theta }}{1-e^{-j\pi \cos\theta }}.$$

After factoring, Equation (24) reduces to:

(25) $$\mathrm{AF}=\left(\frac{e^{-j\frac{M\pi }{2}\cos\theta }}{e^{-j\frac{\pi }{2}\cos\theta }}\right)\frac{\sin\left(\frac{M\pi }{2}\cos\theta \right)}{\sin\left(\frac{\pi }{2}\cos\theta \right)}.$$

**Figure 2:**
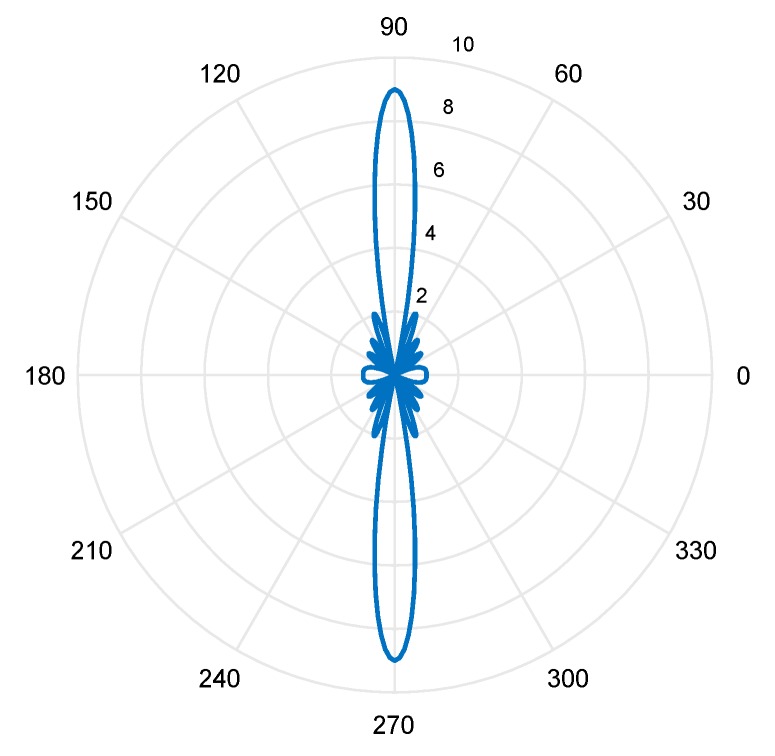
Array factor for uniform linear array (ULA).

The AF magnitude pattern for ULA is shown in Figure 2. The magnitude of the array factor is plotted for an array with M = nine elements. Based on the magnitude of the array factor, the maximum energy is received or transmitted by the array when θ = 90° or when θ = 270°. Introducing weights and varying the orientation of the ULA can manipulate this array factor. Since the array factor is a linear function of the weights, choosing weights is considered a minor procedure. On the other hand, the array factor is a nonlinear exponential function of the element positions; consequently, array geometry optimization is more challenging [34].

Other vital parameters of array factors include the beam width and sidelobe level. The beam width is usually defined as a null-to-null or half-power beam width. The null-to-null beam width is the angular distance between the first nulls around the main beam [34]. The half-power beam width is the angular distance between the half-power points (i.e., 3 dB points on the array factor) around the main beam. The sidelobe level is usually defined as the maximum value of the array factor that is found outside the main beam.

### 4.2. Uniform Circular Array

The planar array geometries can be divided into three other subcategories: circular, rectangular, and square. The circular arrays stand out of the three, as they do not have edge elements. In the absence of edge constraints, the beam pattern of a circular array can be electronically rotated. Moreover, the circular arrays also have the capability to compensate for the effect of mutual coupling by breaking down the array excitation into a series of symmetrical spatial components [35,36].

**Figure 3:**
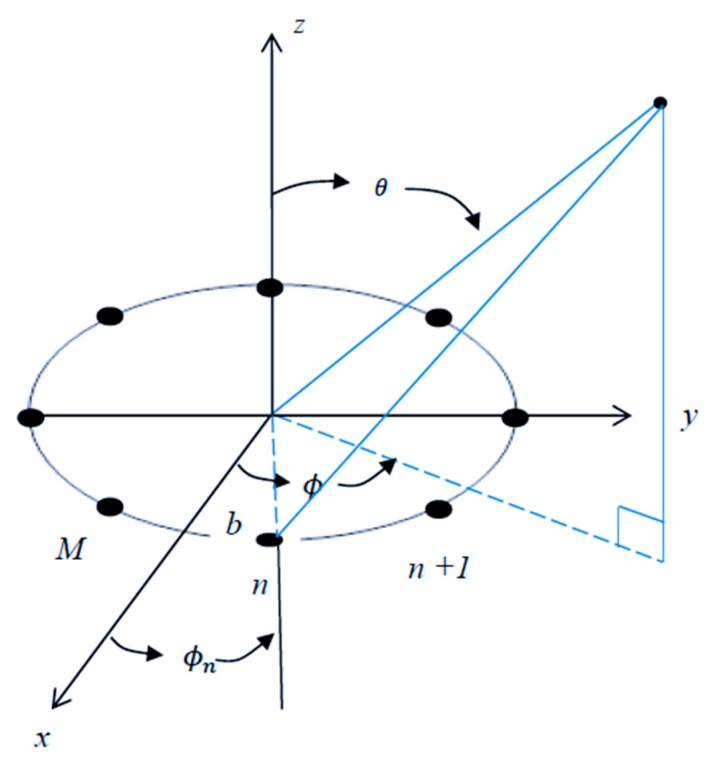
Circular array geometry.

The array factor of a circular antenna array, which is shown in Figure 3, is given by [35]:

(26) $$\mathrm{AF}\left(\theta ,\phi \right)=\overset{M}{\underset{n=1}{\sum }}e^{j\left(kb\sin\theta \cos\left(\phi -\phi_{n}\right)\right)}$$

where $\phi_{n}$ is the angular position of element n, M is the number of antenna elements, b is the radius of the circle, and k is the wavenumber, $k=\frac{2\pi }{\lambda }$.

**Figure 4:**
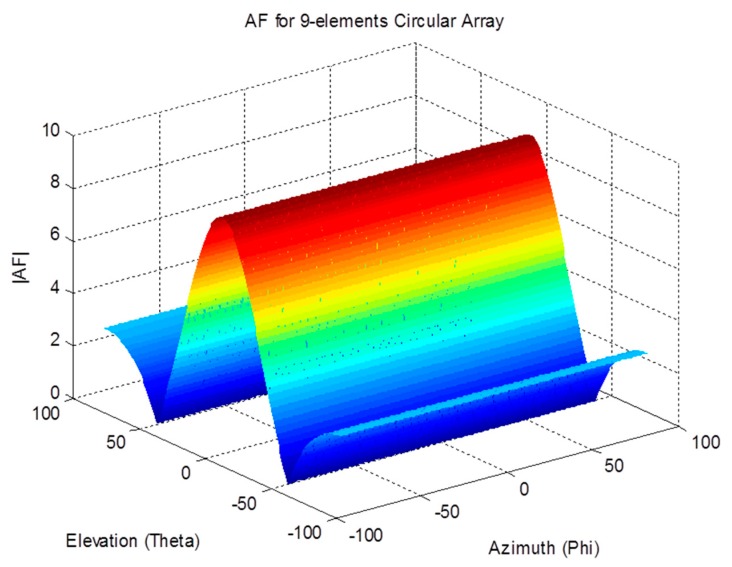
Array factor magnitude for the circular array.

**Figure 5:**
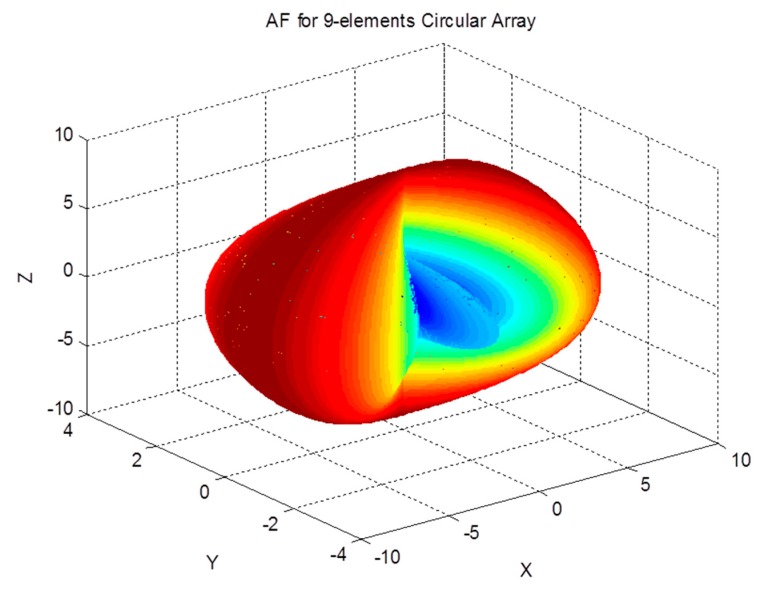
Array factor pattern for the circular array.

**Figure 6:**
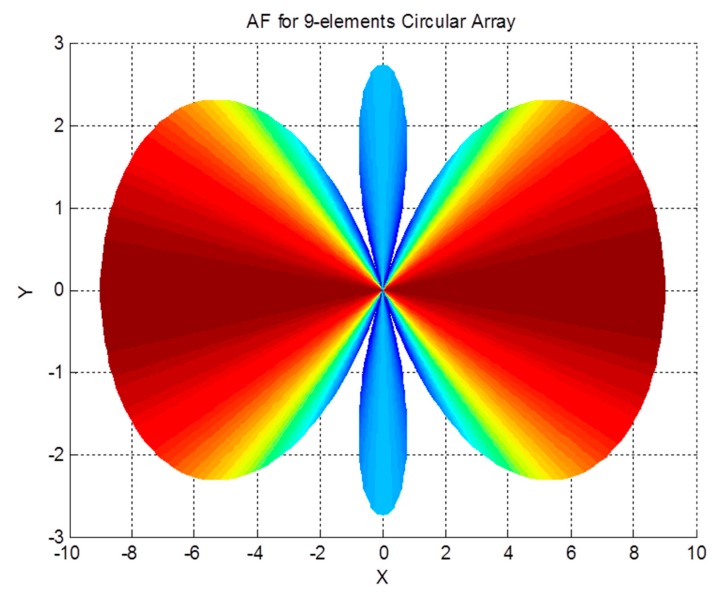
XY-plane view of an array factor pattern for the circular array.

The array factor for a circular antenna array is a function of both spherical angles; therefore, it can filter the received signals based on their azimuth and elevation angles. The effect of the array on the received signal as a function of the angle of arrival can be demonstrated by examining the array factor. The magnitude pattern of the circular array factor is shown in Figure 4, Figure 5 and Figure 6. This pattern is drawn for a circular array of nine elements that are equally spaced on a circular circumference with a radius of $\frac{\lambda }{2}$. The circular arrays configuration does not have the edge effect. It is clear from the AF magnitude shown in Figure 6 that the circular array response is symmetrical around the elevation direction, which enhances the capabilities of circular antenna arrays in beam steering.

## 5. Results and Discussion

**Figure 7:**
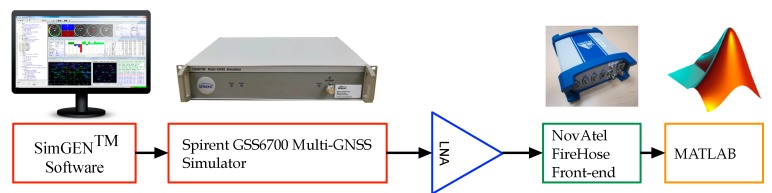
Experimental setup of the Spirent GSS6700 and the Firehose.

The following section demonstrates the results obtained from the different scenarios that were implemented in order to evaluate the performance of the developed high-resolution DOA estimation method for ULA and UCA arrays geometry. The proposed method was tested with single and multiple jamming signals at different jamming-to-signal ratios (JSRs). The GPS L1 signals were obtained from a Spirent GSS6700 simulator and were down-converted and digitized using the Novatel Firehose digital frontend, as shown in Figure 7.

**Figure 8:**
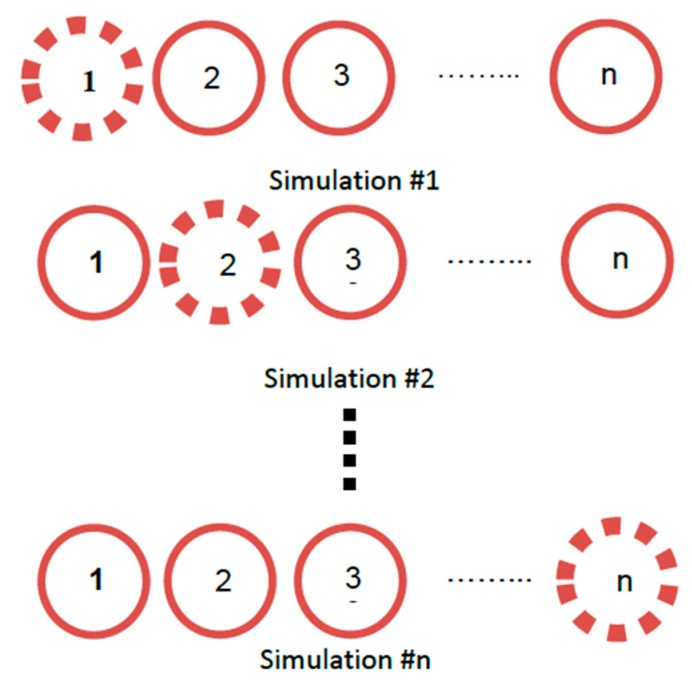
Simulation sequence of an M-element array of GPS antennas [37].

Referring to [37], one radio frequency (RF) front end and a single RF output Spirent GSS6700 simulator running SimGEN^TM^ software are utilized to verify the proposed method. Initially, the desired experimental parameters such as the number of antenna elements, array geometry, GPS signal frequency (i.e., L1, L2 and L5), and consequently antenna element spacing are defined. Upon designating the zero-phase element’s (reference element) location in terms of latitude, longitude, and height, the remaining elements of the array are mapped according to the predefined experimental configuration. Once the locations of all the antenna elements are set, a simulation scenario is created using the simulator software. The simulator software enables the reiteration of the simulation scenario without changing any parameters except for the location of the antenna element in 3D space. Figure 8 illustrates the simulation sequence for a given *M*-element array, where each iteration corresponds to one of the array elements where n=1,2…,M.

The jamming signals frequencies are relative to the GPS signal’s center frequency obtained from digitization and down-conversion. Jamming signals were simulated by adding sinusoidal signals to the DGA output. The simulated jammer frequencies ranged from 100 to 400 Hz. This range ensures that the introduced jamming signals fall within the bandwidth of the DGA output digitized, down-converted GPS L1 signals, which are baseband signals with 1.023 MHz bandwidth.

### 5.1. Results for ULA

- Single Jammer

**Figure 9:**
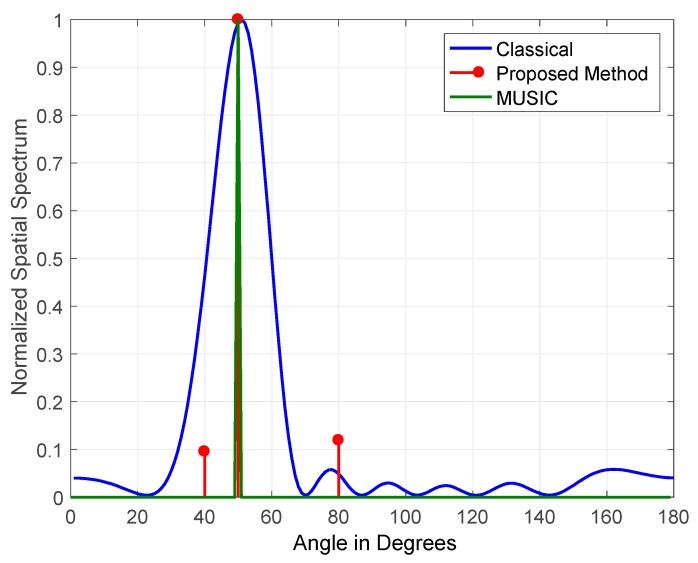
Direction of arrival (DOA) estimation of one jammer at a JSR = 15 dB.

**Figure 10:**
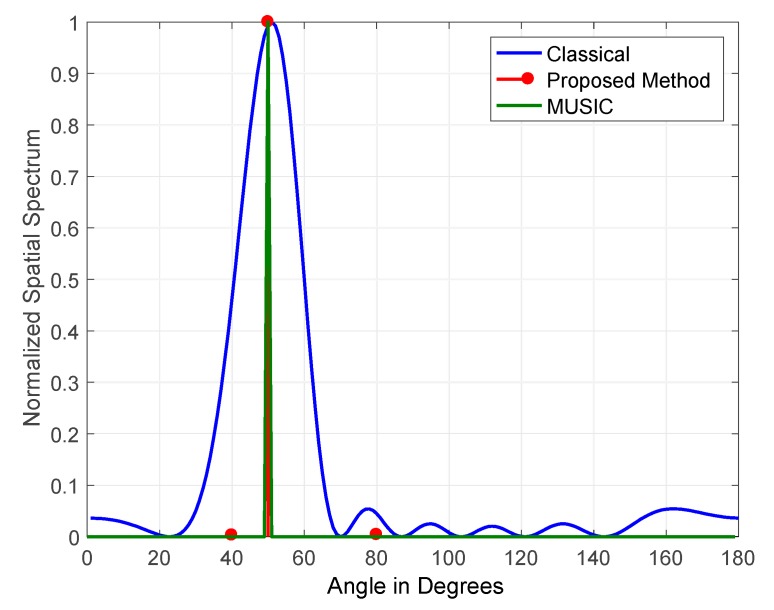
DOA estimation of one jammer at JSR = 45 dB.

The results obtained for a single jammer simulated at a frequency of 100 Hz and arriving at an elevation angle of 50° at a JSR of 15 and 45 dB are shown in Figure 9 and Figure 10, respectively. The figures illustrate the normalized output for the classical, the proposed, and MUSIC DOA estimation methods. The figures demonstrate that all three methods detected the single jammer with high accuracy at JSRs of 15 and 45 dB. When the detection of a single GPS jamming signal is required, the performance of all three methods is satisfactory, as the jammer of interest’s received power is usually high.

The main contribution of the proposed method when estimating the DOA of a single jammer is the high accuracy in the detection of jamming signals’ amplitude, as shown in Figure 10. This is mainly related to the nature of the operation of FOS on which the method is built. It operates by constructing a signal model determined by candidate functions corresponding to the detected signals.

- Multiple Jamming Signals

The performance analysis of FOS versus MUSIC and classical DOA was examined in terms of resolution, tolerance to JSR, and tolerance to the jamming signals coherence. For this purpose, three jamming signals were simulated with frequencies of 100 Hz, 200 Hz, and 300 Hz and arriving at elevations of $50^{{}^{\circ}},\;60^{{}^{\circ}},\;\mathrm{and}\;70^{{}^{\circ}}$, respectively. The tolerance to jamming signals coherency was examined by repeating this scenario with three jamming signals at frequencies of 100, 105, and 110 Hz.

**Figure 11:**
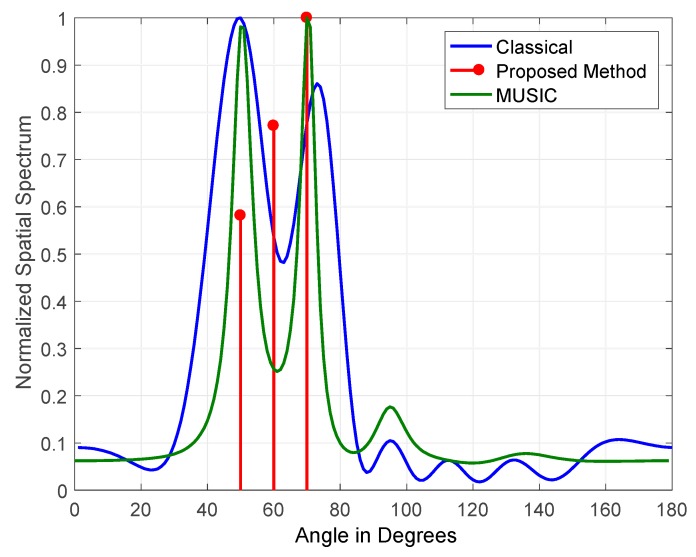
DOA estimation of three jammers at JSR = 15 dB and frequencies of 100 Hz, 200 Hz, and 300 Hz.

**Figure 12:**
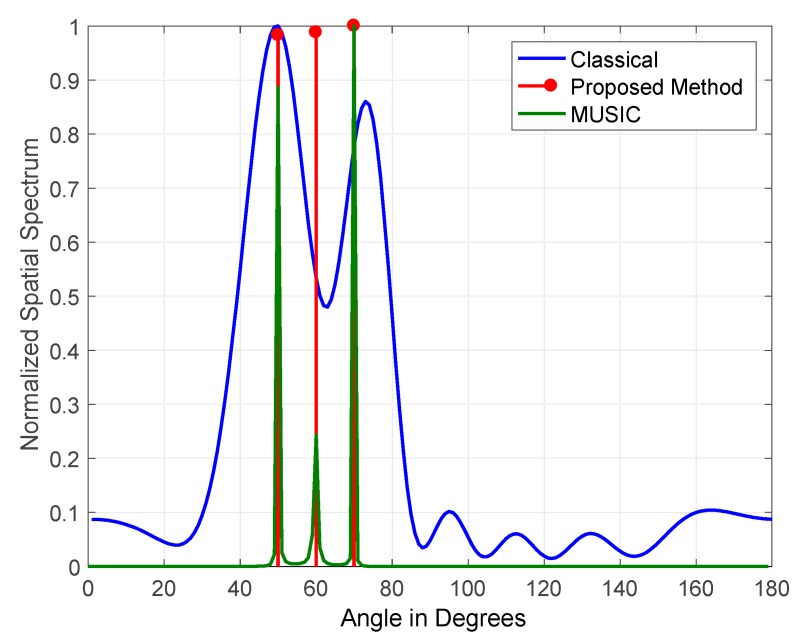
DOA estimation of three jammers arriving at JSR = 45 dB and frequencies of 100 Hz, 200 Hz, and 300 Hz.

The results obtained for three jammers arriving at elevations of (50°, 60° and 70°) JSRs are shown in Figure 11 and Figure 12. The figures indicate the normalized output for classical DOS estimation, MUSIC, and the proposed DOA estimation methods. The JSRs of the jamming signals used in Figure 11 and Figure 12 are 15 and 45 dB, respectively.

The results shown in the above figures demonstrate that the proposed FOS-based method outperformed both the MUSIC and classical methods due to its high tolerance to the variation of JSR and jamming signals coherency. MUSIC detected three jammers at a JSR of 45 dB with zero error in estimated DOAs, but its performance degraded at a JSR of 15 dB as it detected two jammers only. On the other hand, our method’s performance was stable in terms of the number of jammers detected at JSRs of 15 dB and 45 dB, as it detected three jammers accurately with zero error in estimated DOAs. It also degraded slightly at a JSR of 15 dB where its power allocation for detected jammers was not equally divided among the three jammers that were simulated with equal power.

### 5.2. Results for UCA 

The proposed method (FOS) performance was examined using a UCA configuration described earlier, with seven elements equally spaced on the circumference of a circle with a radius of λ2.

- Single Jammer

**Figure 13:**
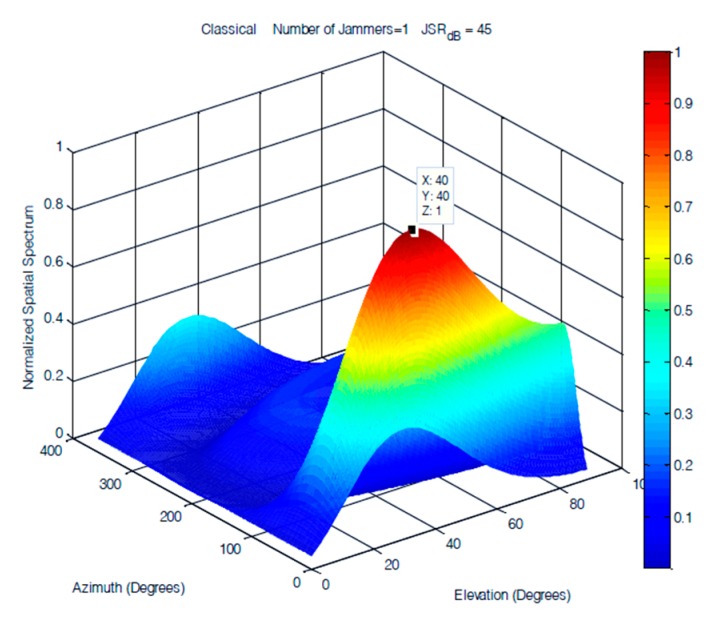
DOA estimation of one jammer using classical DoA at JSR = 45 dB.

**Figure 14:**
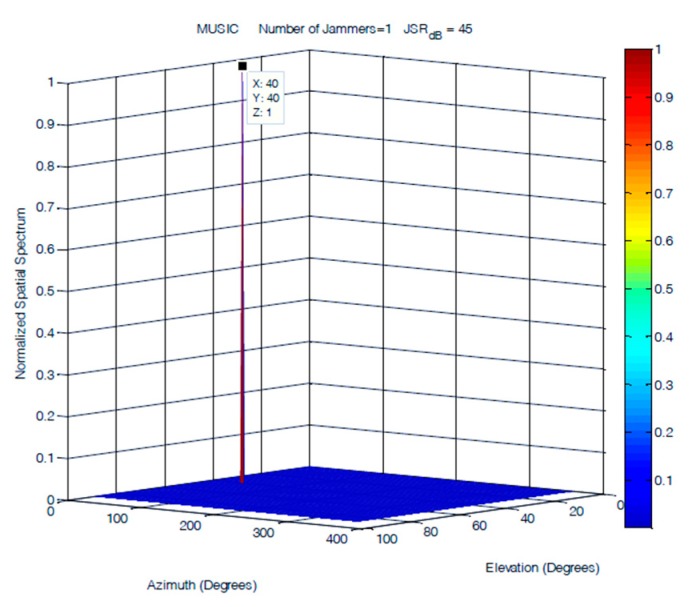
DOA estimation of a single jammer using MUSIC at JSR = 45 dB.

**Figure 15:**
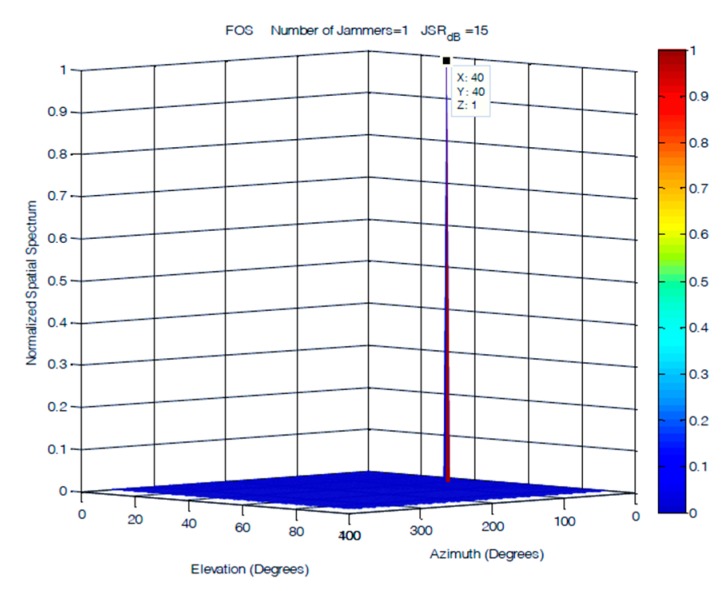
DOA estimation of one jammer using fast orthogonal search (FOS) at JSR = 45 dB.

A single jammer was simulated as a 100 Hz sinusoidal signal arriving at an elevation of 40° and an azimuth of 40°. Figure 13, Figure 14 and Figure 15 demonstrate that the performances of the FOS, MUSIC, and classical DOA in 2D single jammer detection are almost identical and show a clear detection of the jamming signal with zero error in both elevation and azimuth. The advantage of FOS and MUSIC is that their spatial spectrum has much higher resolution compared to that of classical DoA.

- Multiple Jamming Signals

**Figure 16:**
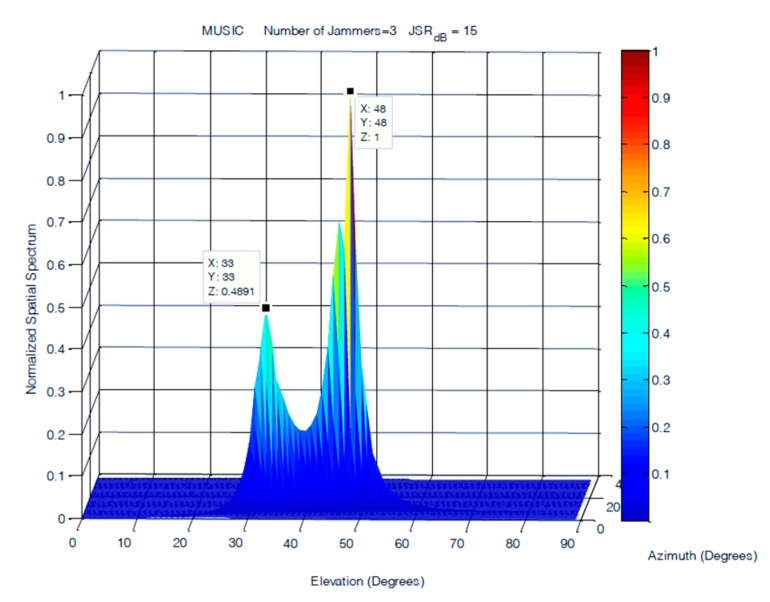
DOA estimation of three closely spaced jammers using MUSIC at JSR = 15 dB.

**Figure 17:**
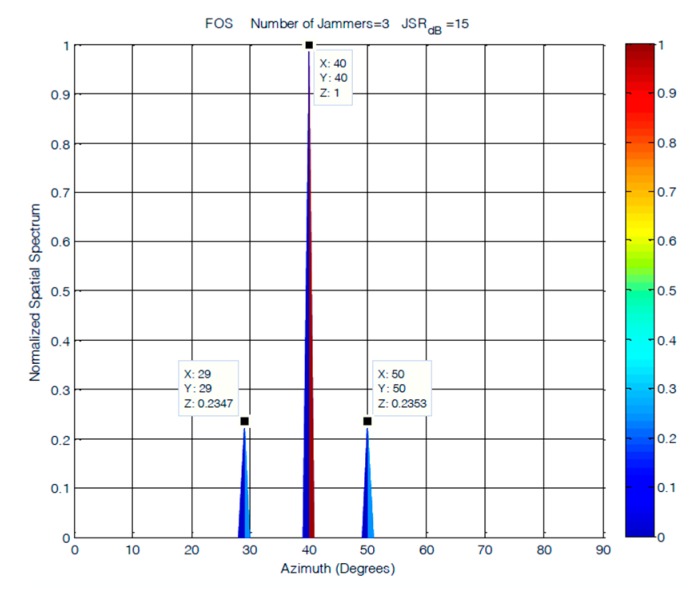
DOA estimation of three closely spaced jammers using FOS at JSR = 15 dB.

In this scenario, three equal power jammers were simulated at frequencies of 100, 200, and 300 Hz with elevations and azimuths separations of 10°. The DoAs of the three jammers were simulated at (30°, 30°), (40°, 40°), and (50°, 50°). This experiment is conducted at JSRs of 15 and 45 dB. The results obtained from the MUSIC and classical DoA estimation methods are shown in Figure 16 and Figure 17. In this scenario, the performance of FOS is compared to MUSIC only, as their performance versus the classical DoA method was already examined for ULA and showed superior performance in all cases over classical DoA.

The results shown in Figure 17 showed steady performance for FOS in terms of the resolution and number of detected jammers for the cases where the JSR was 15 dB. The degradation in FOS was observed in its allocation of power to jamming signals, as it was not accurately allocated for jammers arriving at a JSR of 15 dB. The increase in the level of noise affected FOS performance and caused most of the power to be allocated to the jammer arriving at 40°. MUSIC had much lower accuracy in power allocation for the three jamming signals. The degradation in MUSIC performance was more evident at lower JSR ratios, where MUSIC was able to detect only two jamming sources, as shown in Figure 16. The accuracy of the detected DOA elevation and azimuth of jamming signals at 15 dB JSR are close for both FOS and MUSIC, as shown in Table 1, with a slight increase of 1° in the error of jamming signals detected by MUSIC.

## 6. Conclusions and Future Work

High-resolution DOA estimations for interference detection using the ULA and UCA configurations were studied. A new procedure was utilized to simulate GPS received signals using a single RF output Spirent GSS 6700 GPS simulator and a single RF input Novatel FireHose frontend. Postprocessing validated the GPS signals used. The main contribution of this research is the application of nonlinear signal modeling techniques for GPS jamming detection using ULAs and UCAs. The method employed for jammer DOA estimation is based on FOS. Its performance was evaluated in terms of DOA estimation accuracy, magnitude estimation, and the number of jammers detected at different JSRs. The proposed method was compared to MUSIC and classical DOA estimation. It demonstrated significant improvement in estimating the magnitude of the spatial spectrum when compared to MUSIC and consistently outperformed it. MUSIC only showed similar performance when more powerful jamming signals were applied. The proposed method was more accurate at detecting the amplitudes of the single jammers. In the case of ULA, as for multiple jammers, it outperformed MUSIC by 33% in terms of the number of jammers detected at relatively low JSR. In the case of UCA, as for multiple jammers, the proposed method detected all three jammers, and MUSIC only detected two at relatively low JSRs. Moreover, the proposed method also demonstrated higher accuracy in the detection of the three sources of jammers with slight variations in their amplitude at a relatively low JSR of 15 dB. In addition, the capabilities of the classical method are limited by its low resolution, and MUSIC is much more sensitive to noise and performs worse at lower signal power. Since the proposed method is not optimized for real-time operation, it is limited by its higher computational complexity compared to the classical approach. Nevertheless, the added complexity is justified by the high gains in accuracy. The proposed method will detect a higher number of closely spaced jammers and achieve higher accuracies as the number of array elements increases.

## Figures and Tables

**Table 1:** Results of three closely spaced jammers at JSR = 15dB.

Jammer (s)	1	2	3
Elevation	30°	40°	50°
Azimuth	30°	40°	50°
MUSIC	(33°,33°)	N/A	(48°,48°)
MUSIC (Error)	(3°,3°)	N/A	(2°,2°)
FOS	(29°,29°)	(40°,40°)	(50°,50°)
FOS (Error)	(1°,1°)	(0°,0°)	(0°,0°)
